# Genome-Wide Identification of Natural Resistance-Associated Macrophage Protein (NRAMP) and Expression Analysis Under Heavy Metal Stress in *Sorghum bicolor* L.

**DOI:** 10.3390/plants14172660

**Published:** 2025-08-26

**Authors:** Xiaopan Hu, Xiaoxue Li, Bin Zhu, Lei Gu, Tuo Zeng, Feng Yu, Lang Liu, Hongcheng Wang, Xuye Du

**Affiliations:** 1 School of Life Sciences, Guizhou Normal University, Guiyang 550025, China; 232100100439@gznu.edu.cn (X.H.); 242100100432@gznu.edu.cn (X.L.); 201703008@gznu.edu.cn (B.Z.); leigu1216@gznu.edu.cn (L.G.); zengtuo@gznu.edu.cn (T.Z.); 201711004@gznu.edu.cn (F.Y.); 202409006@gznu.edu.cn (L.L.); 2 National Key Laboratory for Germplasm Innovation & Utilization of Horticultural Crops, College of Horticulture & Forestry Sciences, Huazhong Agricultural University, Wuhan 430070, China

**Keywords:** *Sorghum bicolor* L., *NRAMP* gene, expression analysis, heavy metals

## Abstract

The NRAMP (Natural Resistance-Associated Macrophage Protein) family plays a pivotal role as membrane transporters in plants’ responses to heavy metal stress. This study identified 12 *NRAMP* genes in *Sorghum bicolor* (sorghum) and performed a comprehensive bioinformatics analysis. The *SbNRAMP* genes are distributed across seven sorghum chromosomes. In-depth analyses of gene structure, conserved motifs, collinearity, and phylogeny indicated that the *SbNRAMP* family is divided into three subfamilies, each exhibiting unique structural and motif characteristics. Collinearity analysis suggested that large-fragment duplications, rather than tandem duplications, were responsible for the expansion of the *SbNRAMP* family, resulting in a greater number of genes compared to *Arabidopsis thaliana* and rice. Transcriptome analysis of the aboveground and underground parts of sorghum seedlings under saline–alkali stress revealed that *SbNRAMP5* is a key hub gene exhibiting tissue-specific expression. Furthermore, qRT-PCR analysis following exposure to Cd, Mn, or Zn treatments revealed differential expression among the *SbNRAMP* genes. Subcellular localization predictions indicated that all twelve NRAMPs are primarily located in the plasma membrane, with nine to twelve transmembrane domains, consistent with their function in metal ion transport. Experimental evidence confirmed that *SbNRAMP6* is localized in the plasma membrane. These findings establish a foundation for a deeper understanding of the structure and function of the sorghum *NRAMP* gene family.

## 1. Introduction

Metal ions play a variety of crucial roles in plants, significantly influencing their metabolic processes [1]. For instance, copper (Cu) deficiency can result in stunted growth and diminished plant stature [2]. Meanwhile, magnesium (Mg) deficiency adversely affects chloroplast formation, thereby impairing photosynthesis [3]. Zinc (Zn) is essential for regulating auxin metabolism and plant growth [4]. Manganese (Mn) is vital for numerous physiological activities, including photosynthesis, flavonoid synthesis, and the activation of enzyme hormones [5]. Furthermore, certain transition metals, such as iron (Fe), zinc (Zn), and copper (Cu), act as biocatalysts and structural cofactors, thereby enhancing the diversity of protein structures and functions through their binding to proteins [6]. Conversely, excessive concentrations of metals can be detrimental to plants. High levels of copper can damage the root apical meristem, inhibit root elongation, and reduce lateral root formation. Similarly, excessive zinc can disrupt the structure of chloroplasts and diminish the activity of chlorophyll synthase [7]. Certain heavy metals, such as cadmium (Cd) and manganese (Mn), exhibit toxic effects on plant growth and development [8]. In recent years, industrial development has resulted in significant emissions of heavy metals into the environment. Concurrently, heavy metals introduced during the production and use of chemical nitrogen fertilizers have emerged as a critical source of soil pollution [9]. These pollutants not only diminish crop yields but also pose a serious threat to human health [10].

*Sorghum bicolor* (Hongyingzi) is a monocotyledonous plant belonging to the genus *Sorghum* within the family *Poaceae*. It is characterized as a typical diploid plant with a chromosome number of 2n = 20 [11,12]. As one of the world’s top five cereal crops in terms of yield and area cultivated, sorghum is a unique multi-purpose crop and extensively cultivated in regions subject to severe conditions, including drought and saline–alkali soils, due to its remarkable resistance to abiotic stresses [13]. Based on its domestication pathways and utilization characteristics, it can be categorized into three types, grain sorghum, energy sorghum, and silage sorghum, all of which hold significant breeding and economic value [14]. Due to its remarkable resistance, sorghum effectively alleviates various abiotic stresses, such as salinity and drought. Consequently, it has emerged as a viable phytoremediation strategy to mitigate soil heavy metal pollution [15]. Compared to traditional remediation methods, phytoremediation technology offers significant advantages, including environmental sustainability and the absence of secondary pollution. This technology is considered one of the key adaptive strategies for addressing soil pollution and degradation, thereby enhancing the quality and resilience of cultivated land [16]. Consequently, an in-depth exploration of the absorption, transport, and degradation mechanisms of heavy metals by sorghum, a gramineous plant, not only enhances soil remediation efficiency but also holds significant practical implications for reducing heavy metal residues in agricultural products.

NRAMPs (Natural Resistance-Associated Macrophage Proteins) play a crucial role in the transmembrane transport of metal ions in plants. This protein family can significantly mitigate the adverse effects of heavy metals on plant growth [17]. Further exploration has revealed that the *NRAMP* gene family plays a crucial role in the uptake and transport of nutritionally essential divalent cations, including Fe^2+^, Mn^2+^, and Zn^2+^. Notably, these transporters also facilitate the absorption and transport of Cd^2+^ in plants [18]. Studies have demonstrated that the *NRAMP* gene family is widely distributed across animals, plants, and bacteria, with a notable prevalence in plants. There are substantial variations in the number of *NRAMP* gene family members among different plant species. For instance, *Oryza sativa* contains 7 *NRAMP* gene family members [19], *Setaria italica* has 12 [20], and *Brassica napus* possesses 22 [21], while *A. thaliana* only has 6 *NRAMP* gene members [22]. Research indicates that the *NRAMP* gene family is vital for various plant species. For instance, *AtNRAMP1* functions as a high-affinity manganese transporter; its loss of function significantly inhibits plant growth under manganese stress [23]. In rice, *OsNRAMP1*, a divalent metal transporter primarily responsible for the uptake of ions such as Fe and Mn, has also been identified as a critical regulator of Cd accumulation. Gene knockout experiments have demonstrated that the deletion of *OsNRAMP1* significantly reduces the uptake and transport of cadmium in rice [24]. In *Arachis hypogaeas*, Mn or Zn deficiency in roots and stems strongly induced the transcription level of *AhNRAMP1* [25]. In *Triticum aestivum*, *TaNRAMP5* encodes a transporter for Mn and Fe. Its overexpression enhances Cd tolerance in both wheat and tobacco, likely due to the competition between Cd and essential metals for transport or the alteration of metal homeostasis [26]. Moreover, studies have shown that salt treatment can affect the homeostasis of metal ions in plants and induce the expression of *NRAMP* genes, and it is accompanied by the accumulation of iron, zinc, and copper ions in the leaves [27].

In this study, we performed a comprehensive genome-wide identification and systematic characterization of the *NRAMP* gene family in sorghum using bioinformatics approaches. The identified *SbNRAMP* members were subjected to multi-dimensional characterization encompassing gene structure characteristics, conserved motif analysis, phylogenetic relationships, subcellular localization prediction, and tissue expression patterns. Furthermore, we systematically studied the expression profile of the *SbNRAMP* genes in response to metal stresses. Sorghum seedlings were treated with Mn^2+^, Zn^2+^, or Cd^2+^, and the gene expression profiles were subsequently analyzed. This comprehensive analysis provides novel insights into the functional divergence of *SbNRAMP* genes, establishing a theoretical foundation for the identification of key candidate genes involved in sorghum molecular breeding and phytoremediation potential.

## 2. Results

### 2.1. Identification of SbNRAMP Gene Family Members and Analysis of Their Physicochemical Properties

Within the sorghum genome, 12 *SbNRAMP* genes were identified utilizing TBtools and the Pfam online tool. These genes were designated based on their chromosomal positions, and their physicochemical properties were characterized (Table 1). SbNRAMPs vary in length from 466 to 1236 amino acids, with molecular weights ranging from 50.75 to 134.71 kDa. Theoretical isoelectric points range from 4.89 to 8.52, with only SbNRAMP1, 6, 10, and 12 exceeding a pI of 7, indicating that the majority of family members are acidic. The instability for SbNRAMP3, 8, and 10 exceeds 40, suggesting that these proteins are less stable, while the remaining proteins are generally stable, with instability coefficients below 40. The hydrophilicity index of SbNRAMP8 is negative, whereas the indices for the others are positive, indicating that most SbNRAMPs are hydrophobic.

### 2.2. SbNRAMP Gene Members Were Mapped on Seven Chromosomes

**Figure 1:**
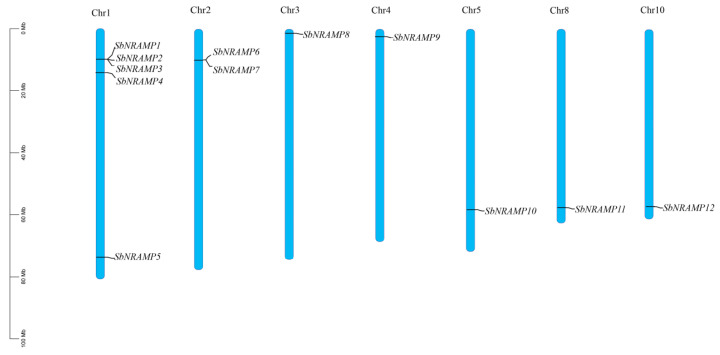
The location of *SbNRAMP* genes on chromosome. The chromosome number is above, with ‘Chr’ denoting the respective chromosome. The *SbNRAMP* genes is located on the right side of the chromosome. The scale on the left represents the chromosome length in megabases (Mb). *SbNRAMP* chromosomal positions were extracted from sorghum GFF files and visualized using TBtools v2.085.

Chromosomal mapping identified the precise locations of *SbNRAMP* gene family members in sorghum (Figure 1); the distribution is uneven across the seven chromosomes. Chromosome 1 contains the highest number of *SbNRAMP* genes (five), while chromosome 2 harbors two genes. Additionally, one *SbNRAMP* gene is present on chromosomes 3, 4, 5, 8, and 10. Most genes are situated near chromosome ends, away from centromeric regions. Given that centromeres and their flanking regions are hotspots for genomic rearrangement and sequence variation [28,29], the observed peripheral localization suggests that *SbNRAMP* genes have likely maintained structural and functional stability over their evolutionary history by avoiding these dynamic chromosomal regions.

### 2.3. The Evolutionary Relationships of the Sorghum NRAMP Family in Comparison to Homologous Genes from Various Plant Species

**Figure 2:**
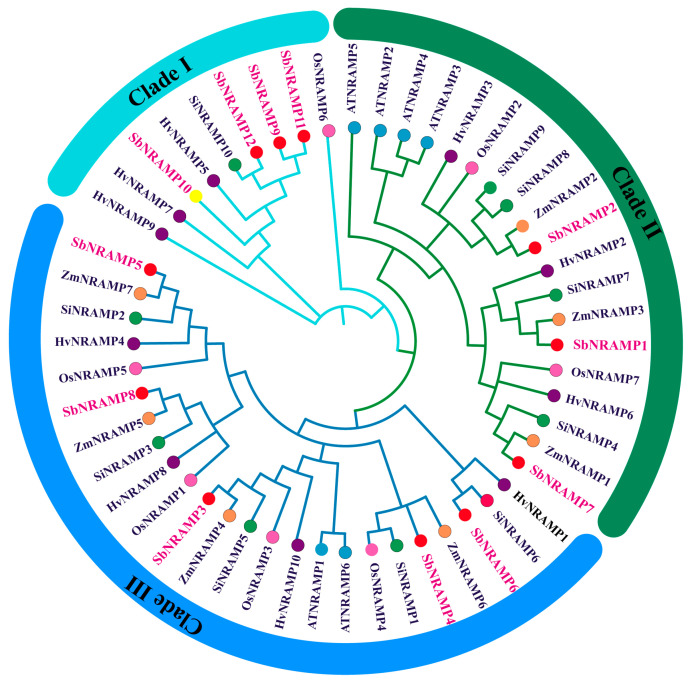
Phylogenetic analysis of 52 NRAMPs in *Oryza sativa*, *Arabidopsis thaliana*, *Setaria italica*, *Hordeum vulgare*, *Zea mays*, and *Sorghum bicolor*. The proteins were divided into three subfamilies. The members of the SbNRAMP family were marked with red fonts, and different species were distinguished by branch nodes with different colors. Note: At represents *Arabidopsis thaliana*, Si represents *Setaria italica*, Hv represents *Hordeum vulgare*, Os represents *Oryza sativa*, and Zm represents *Zea mays*. The neighbor-joining tree was created using MEGA11.0 (bootstrap value = 1000). NRAMP sequences can be accessed from the NCBI database.

To investigate the evolutionary relationships of the sorghum *NRAMP* gene family with those from other species, we conducted a phylogenetic analysis utilizing NRAMP sequences derived from *Oryza sativa*, *Arabidopsis thaliana*, *Setaria italica*, *Hordeum vulgare*, *Zea mays*, and *Sorghum bicolor* (Figure 2); the resulting phylogenetic tree comprises three distinct subfamilies, with the 12 SbNRAMPs evenly distributed among them. Further analysis reveals that the SbNRAMPs are most closely related to those found in *Z. mays.* Several SbNRAMPs exhibit close phylogenetic branches with their maize counterparts, specifically, ZmNRAMP2-SbNRAMP2, ZmNRAMP3-SbNRAMP1, ZmNRAMP7-SbNRAMP5, ZmNRAMP1-SbNRAMP7, ZmNRAMP6-SbNRAMP4, ZmNRAMP5-SbNRAMP8, and ZmNRAMP4-SbNRAMP3. The remaining proteins demonstrate distinct genetic affinities: SbNRAMP6 is closely related to SiNRAMP6 from *S. italica*; SbNRAMP9, SbNRAMP11, and SbNRAMP12 exhibit close relationships with SiNRAMP10; and SbNRAMP10 shows a strong association with HvNRAMP5 from *H. vulgare*.

### 2.4. Comparative Analysis of SbNRAMP Phylogenetic Relationships, Gene Structures, and Conserved Motifs in Sorghum bicolor

**Figure 3:**
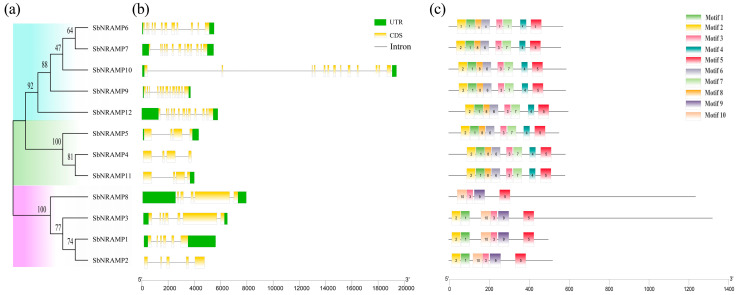

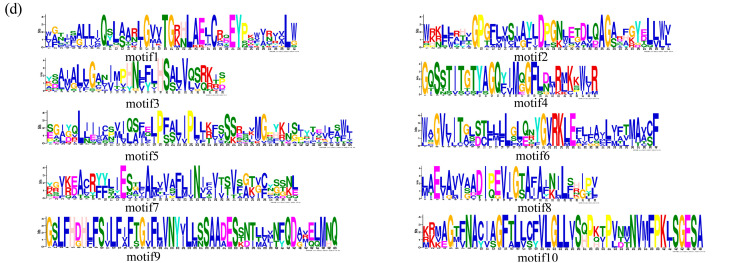
Conservation motif and gene structure analysis of *SbNRAMP* genes according to the phylogenetic relationship. (**a**) Phylogenetic tree: The maximum likelihood tree was constructed using MEGA11.0 software, based on SbNRAMP sequences sourced from Phytozome v13 and NCBI, with 1000 bootstrap replicates; blue, green, and purple backgrounds represent subfamilies I, II, and III. (**b**) Gene structure: Orange and green squares represent exons and untranslated regions (UTRs), respectively. Black solid line represents intron; the following bar scale represents the gene length. (**c**) Conserved motif: Motifs (1 to 10) were identified using TBtools and are represented by colored rectangles; the black solid line represents the sequence outside the pattern; the bar scale represents the number of amino acids. (**d**) Identification of conserved amino acid residue sequences: Sequence logos depict conserved amino acid residue sequences (motifs) identified using the MEME online tool with the number of motifs set to 10 and other parameters at default settings. The *X*-axis indicates the positions of various amino acids, while the *Y*-axis represents the corresponding positional values of these amino acids.

Phylogenetic analysis of the SbNRAMP sequences classified the 12 proteins into three subfamilies (Figure 3a). Subfamily I comprises SbNRAMP6, 7, 9, 10, and 12; subfamily II includes SbNRAMP4, 5, and 11; and subfamily III consists of SbNRAMP1, 2, 3, and 8. Furthermore, we observe that the classification of the three subfamily members diverges from the results obtained through phylogenetic analysis (Figure 2). This discrepancy arises because the 12 genes exhibit varying degrees of protein structural similarity with other species, leading to differing classification outcomes compared to those derived from phylogenetic methods. Structural analysis revealed considerable diversity among these genes (Figure 3b), with intron numbers ranging from 3 to 12 and exon counts varying from 4 to 13. Members of the same subfamily exhibit notable conservation of gene structural features, particularly in terms of highly consistent exon numbers and arrangement patterns. The introns and exons present in each gene are detailed in Table 2. Conserved motif analysis (Figure 3c) identified 10 motifs, with each SbNRAMP containing 4 to 8 motifs. Subfamilies I and II display eight motifs each, indicating structural similarity, while in subfamily III, SbNRAMP8 protein contains only four motifs, and the others each possess six. The amino acid composition and positional frequencies of the motifs are examined in detail by us (Figure 3d).

### 2.5. Analysis of SbNRAMP Cis-Acting Elements

**Figure 4:**
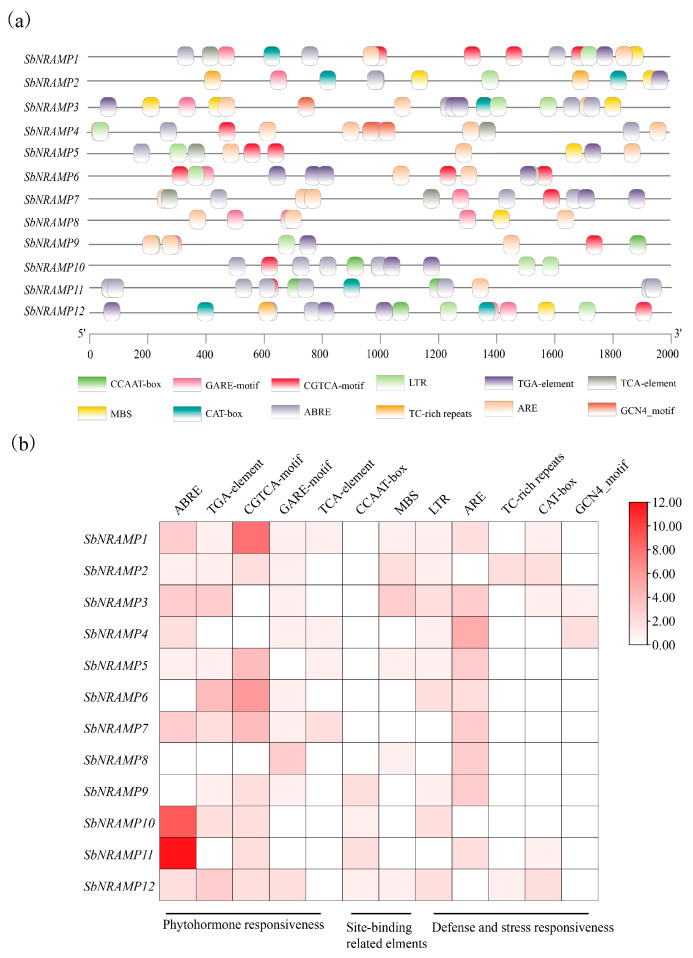
Promoter *cis*-acting elements. (**a**) Identification of the *cis*-acting elements in the promoter of *SbNRAMP* genes. (**b**) A heatmap was utilized to visualize the types and counts of identified *cis*-acting elements within promoter regions for better visualization and understanding of their distribution. Promoter sequences (2 kb upstream of ATG) from *SbNRAMP* genes were analyzed in PlantCare for *cis*-acting elements. Target elements were filtered in Microsoft Excel 2019 and visualized using TBtools.

The analysis of the 2000 bp upstream promoter regions of the *SbNRAMP* genes identified twelve distinct *cis*-elements (Figure 4a). These promoters are enriched with response elements for abscisic acid, gibberellin, and drought stress, alongside core promoter motifs such as TATA and CAAT boxes. The *cis*-elements were categorized into five groups: (i) hormone response (ABRE, TGA, CGTCA, GARE, and TCA), (ii) light response (not displayed), (iii) stress response (LTR, ARE, TC-rich repeats, CAT-box, and GCN4-motif), (iv) regulation of plant development (CCAAT-box and MBS), and (v) others (not displayed). This distribution indicates that the *SbNRAMP* gene family is involved in hormone signaling, drought and stress responses, light-mediated regulation, and various physiological processes essential for sorghum growth, metabolism, and environmental adaptation. The diversity of *cis*-elements among the promoters suggests potential functional diversification within the *SbNRAMP* gene family. Heatmap analysis revealed a widespread distribution of hormone-responsive elements, transcription factor binding sites, and defense- and stress-related elements across the promoters (Figure 4b).

### 2.6. Intraspecific and Interspecific Collinearity Analysis of SbNRAMP

**Figure 5:**
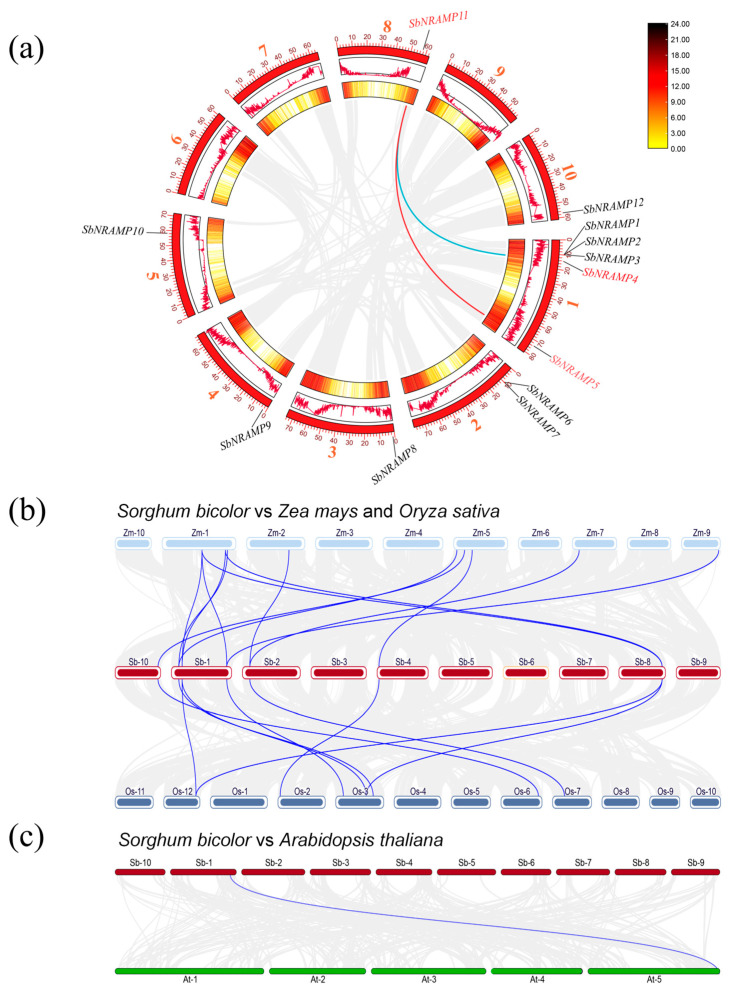
Collinearity analysis of *SbNRAMP* gene family. (**a**) Interspecific synteny: The red numbers on the outermost ring correspond to the chromosome numbers, while the red and blue lines in the inner ring indicate distinct replication events of the *SbNRAMP* genes. Furthermore, the gray lines illustrate the collinear blocks within the plant genome. (**b**) Synteny analysis of the sorghum NRAMP family in comparison with maize and rice. (**c**) Synteny analysis of sorghum and *A. thaliana* NRAMP family: The blue lines in the figure are the common genes between sorghum, *A. thaliana*, rice, and maize, and the gray lines represent the collinear blocks of the plant genome. Note: Zm represents maize, Os represents rice, and At represents *A. thaliana*. Data organization was performed using Microsoft Excel 2019. Visualization was generated with Tbtools.

The collinearity analysis of 12 *NRAMP* family members in sorghum revealed two fragment replication events involving *SbNRAMP11* with *SbNRAMP5* and *SbNRAMP4* (Figure 5a). The *SbNRAMP11* gene underwent two substantial replication events in different orientations, leading to these events occurring on distinct chromosomes. This suggests that the *SbNRAMP11* gene has experienced significant replication during evolution. In comparison to rice and *A. thaliana*, the number of *SbNRAMP* gene family members in sorghum has increased, which may be attributed to the extensive replication events of *SbNRAMP* gene family members.

Collinearity analysis revealed syntenic relationships between sorghum and two monocot species. In the comparison between sorghum and maize (Figure 5b), 12 collinear gene pairs were identified, distributed across different chromosomes of maize. Additionally, in the case of sorghum and rice, nine collinear gene pairs were found on rice chromosomes 2, 3, 6, 7, and 12 (Figure 5c) in the cruciferous dicotyledonous plant *A. thaliana*, and for sorghum, a collinear gene pair on chromosome 5 of *A. thaliana* was identified. This finding indicates that sorghum exhibits a closer evolutionary relationship with *NRAMP* genes in maize and rice than with those in *A. thaliana*.

### 2.7. Subcellular Localization Prediction, Transmembrane Domain Prediction, Protein Structure, and Sequence Analyses of SbNRAMP

Subcellular localization and transmembrane domain analyses were conducted to evaluate the roles of SbNRAMPs in heavy metal transport. According to Table 2, SbNRAMP3 is predicted to localize to the plasma membrane and nucleus, while SbNRAMP8 is expected to be found in the plasma membrane as well as in the chloroplast. The remaining members of the SbNRAMP gene family (*SbNRAMP1*, *2*, *4*, *5*, *6*, *7*, *9*, *10*, *11*, *12*) are only associated with the plasma membrane. Given that the majority of genes are located in the plasma membrane (10 out of 12), it is likely that most SbNRAMPs are primarily involved in facilitating ion transport related to membrane functions.

Secondary structure analysis revealed that all proteins comprise four types of secondary structures, with α-helix and random coil being predominant. Notably, SbNRAMP3 and SbNRAMP8 exhibit a higher content of random coils relative to α-helices, distinguishing them from other family members and suggesting potential functional divergence (Table 2).

**Figure A1:**
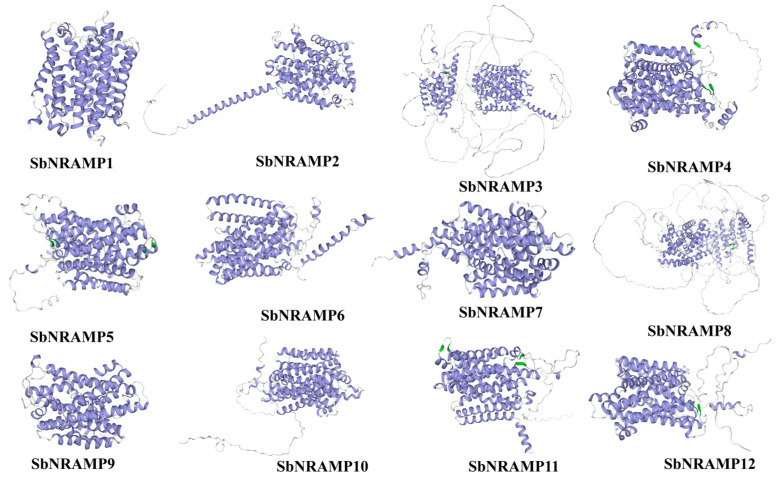
Protein tertiary structure prediction of SbNRAMP gene family members. Purple represents the α-helix structure, green for β-strands, and grey for loops and coils. Tertiary structures were modeled and visualized using SWISS-MODEL with default parameters.

**Figure A2:**
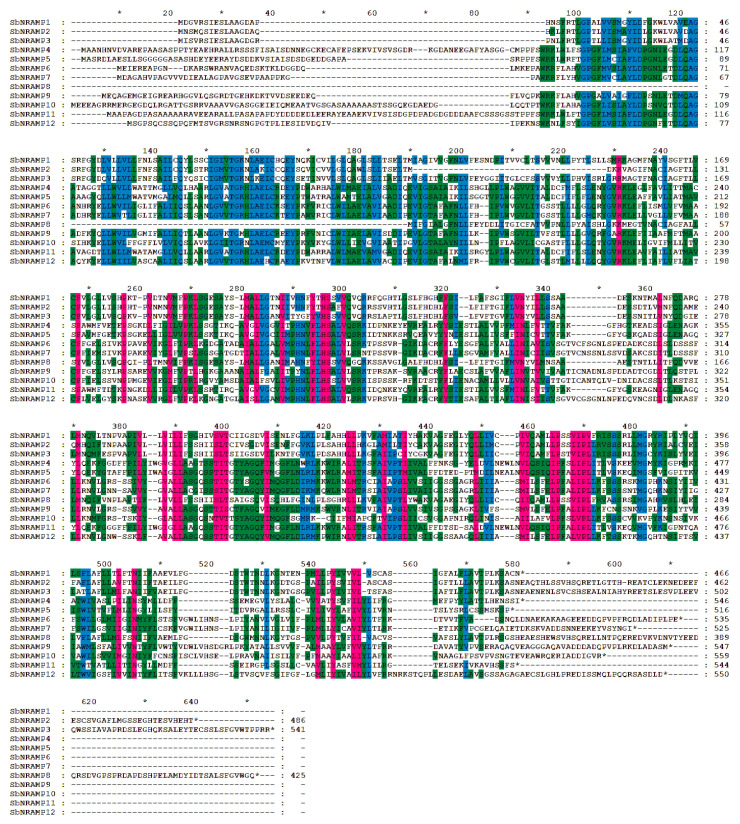
The protein sequence alignment of SbNRAMP; red, blue, and green, respectively, indicate that the amino acid conservation is 100%, greater than or equal to 80%, and greater than or equal to 60%. The asterisks (*) above the alignment denote fully conserved residues across all sequences. Protein sequences aligned with MEGA 11.0 and visualized using GeneDoc.

To elucidate the spatial structure of SbNRAMPs, we employed a comparative modeling approach using *A. thaliana* as a reference to analyze the tertiary structure of 12 SbNRAMP family members (Figure A1). The results of our analysis highlight the distinctions among different subfamilies as well as the similarities among members of the same subfamily. Furthermore, in conjunction with the secondary structure analysis, we can confirm that the results for comparative modeling are reliable. Notably, all members exhibit a prominent helical structure that occupies a central position, accompanied by a limited number of folding fragments. The ends of multiple SbNRAMP members exhibit irregular curls, and the structural similarity among subfamily members is pronounced. In subfamily III (Figure 3a), SbNRAMP3 and SbNRAMP8 are particularly noteworthy, as they possess a substantial number of random coils at both ends of the proteins. Furthermore, the conserved motif maps of these two genes indicate that they contain an extended CDS structure. The protein sequences of 12 members of the *NRAMP* gene family in sorghum were compared and analyzed (Figure A2), with a subsequent examination of their amino acid frequencies. The results indicated that the SbNRAMPs contain multiple conserved regions.

### 2.8. Tissue Expression Analysis of SbNRAMP

**Figure 6:**
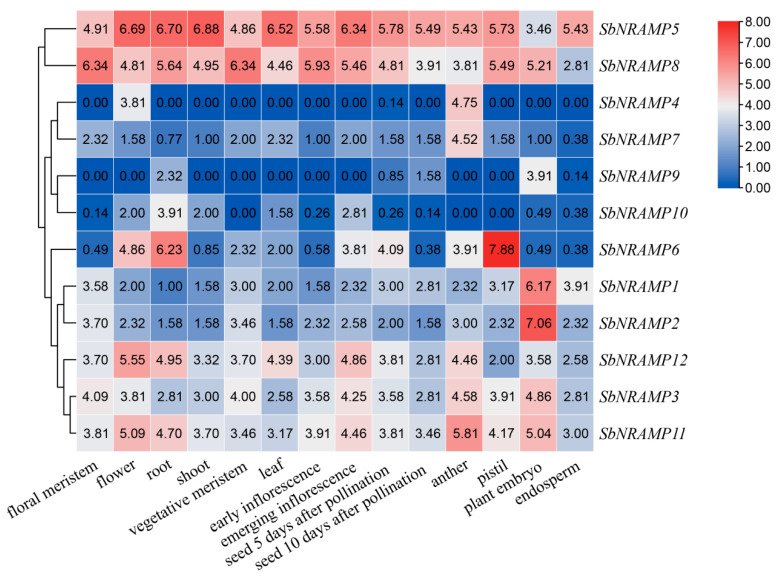
Tissue expression heatmap of *SbNRAMP* gene family members. Gene expression is expressed in log_2_ ^FPKM^. The abscissa represents various tissues of sorghum, while the left side of the ordinate displays clustering based on expression levels. The scale on the right side indicates that blue corresponds to low expression and red to high expression. Each grid cell contains a specific expression value. Data source: Sorghum Functional Genomics Database. Visualization was generated with Tbtools.

In the sorghum database, we obtained expression data for 12 *SbNRAMP* genes across 14 tissues under standard growth conditions (28 °C during the day and 25 °C at night, with a 14-h light/10-h dark cycle and 60–65% relative humidity). Utilizing this data, we generated a heatmap to analyze the differential expression patterns (Figure 6). Based on the expression levels of the 12 genes in various tissues, we categorized these genes into two groups: a high expression group (expressed in ≥2 tissues) and a low expression group (expressed in <2 tissues). A log_2_ ^FPKM^ value of ≥4 was established as the threshold for high expression. Our findings revealed that *SbNRAMP5* exhibited high expression in 13 out of 14 tissues, while *SbNRAMP8* displayed high expression in 11 out of 14 tissues. Notably, *SbNRAMP5* was most significantly expressed in roots, shoot, and flowers, whereas *SbNRAMP8* peaked in floral and vegetative meristems. Additionally, *SbNRAMP3*, *6*, *11*, and *12* also demonstrated high expression across multiple tissues. Specifically, *SbNRAMP12* and *SbNRAMP11* were highly expressed in five tissues, *SbNRAMP3* was expressed in five tissues, and *SbNRAMP6* was expressed in four tissues. Conversely, *SbNRAMP1*, *2*, *4*, *7*, *9*, and *10* were classified as low expression genes, with *SbNRAMP1*, *2*, *4*, and *7* showing high expression in only one tissue, and *SbNRAMP9* and *10* exhibiting low expression across all 14 tissues. This finding suggests that the expression of *NRAMP* gene family members in sorghum is tissue-specific. Most genes are highly expressed in plant embryos, anthers, roots, flowers, and leaves, whereas expression levels are low in endosperm and seeds 5 and 10 days after pollination stages.

### 2.9. WGCNA for Identification of Hub Genes

**Figure 7:**
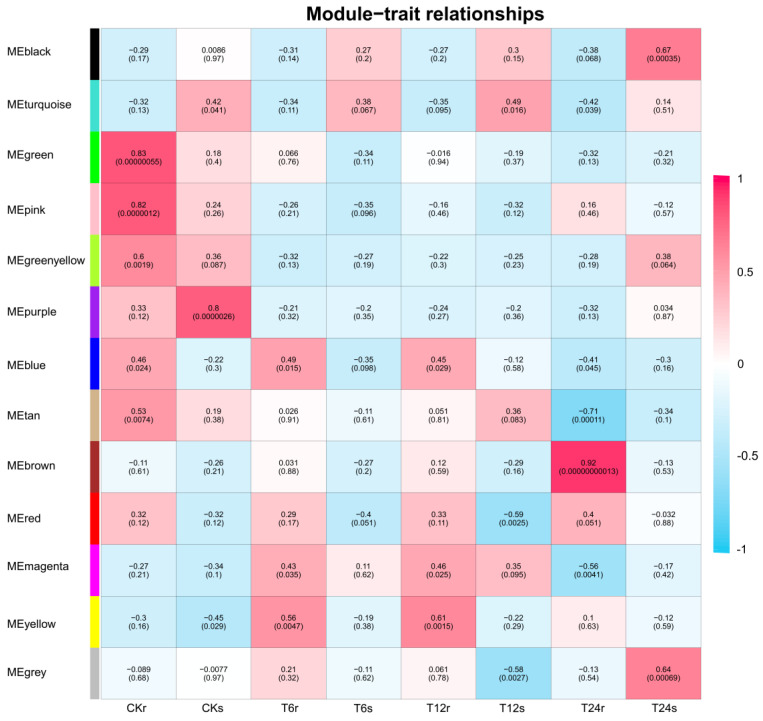
Screening of key candidate gene *SbNRAMP5* from the *SbNRAMP* gene family under saline–alkali stress. Module–trait associations under saline–alkaline stress at 0h, 6 h, 12 h, and 24 h. The colors, ranging from blue through white to red, indicate low to high correlations. CK represents control samples, T represents street time, and s and r represent the shoots and roots of sorghum samples. Visualization was generated with Tbtools.

**Figure 8:**
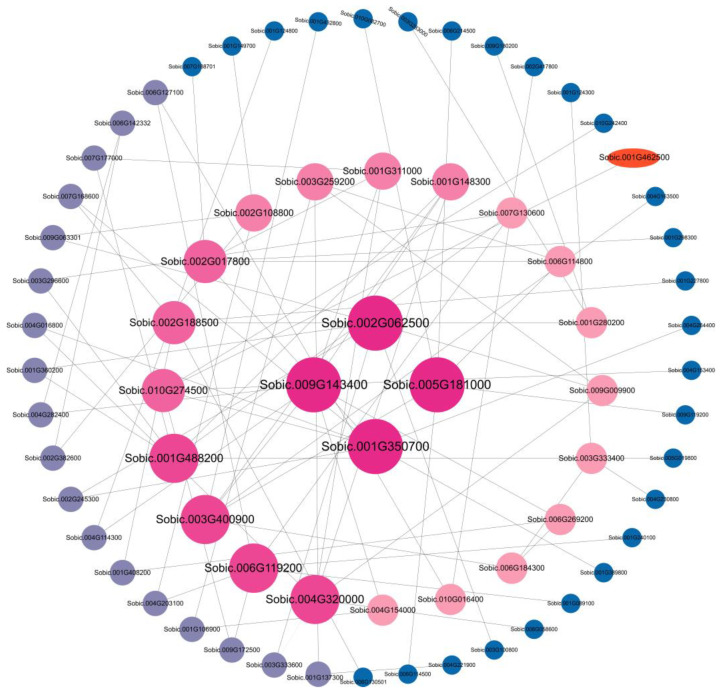
The protein–protein interaction (PPI) network of differentially expressed genes (DEGs) under T6h/yellow 0.56 conditions is presented. In this network, nodes represent proteins, while edges indicate interactions between them. The different colors represent the degree centrality of each node, which is the number of interactive connections a protein has. The red ellipse in the outermost layer highlights the gene *SbNRAMP5* (ID: Sobic.001G462500). The networks were visualized using Cytoscape (version 3.9.1).

To identify potential gene modules associated with abiotic stress (saline–alkaline), we conducted a weighted gene co-expression network analysis (WGCNA) on the transcriptome data of sorghum seedlings subjected to saline–alkaline stress (150 mM NaHCO_3_, pH = 8.0, treated for 0, 6, 12, and 24 h) (Figure 7). After excluding low-expression genes (specifically those with FPKM < 1) from the expression matrix, WGCNA was performed on the filtered transcriptome data. A total of 13 distinct gene modules were identified, each represented by a unique color tile in the adjacency matrix. Upon examining the positive correlation coefficients, the key gene *SbNRAMP5* (Sobic.001G462500) was identified within the positive correlation module of the co-expression network (T6h/yellow 0.56). Furthermore, protein–protein interaction (PPI) analysis revealed that *SbNRAMP5* interacted with other genes within this module, indicating its significant role in abiotic stress response (Figure 8).

### 2.10. Cd, Mn, or Zn Induced the Expression of SbNRAMP

**Figure 9:**
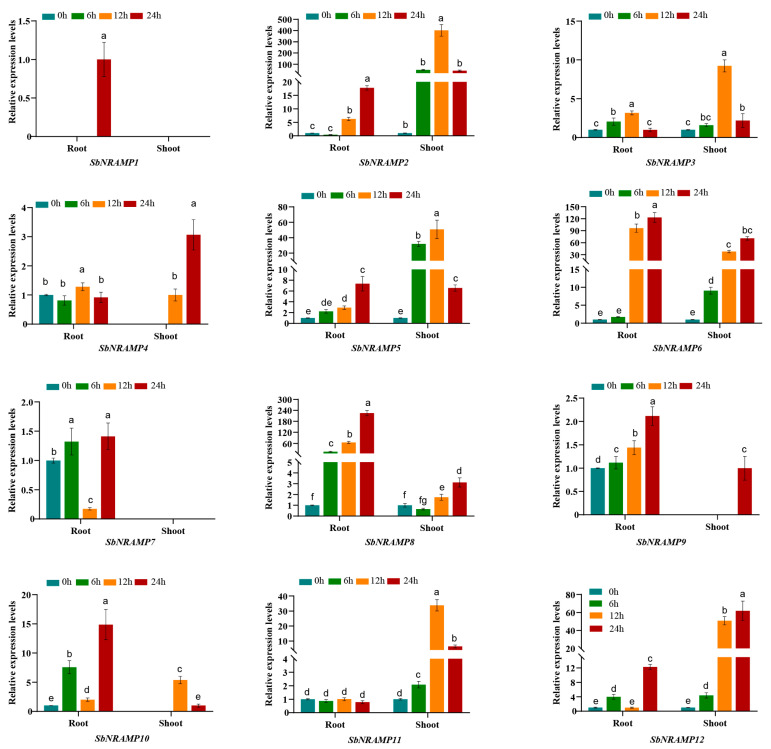
Expression patterns of *SbNRAMP* genes in sorghum seedling roots and shoots under cadmium (Cd) stress. Transcript levels of *SbNRAMP* genes in two-leaf- and one-heart-stage sorghum seedlings after 6, 12, and 24 h of treatment with 100 μmol/L CdCl_2_ were analyzed. Gene expression levels were normalized using the sorghum *actin* gene (*SbACT*) as the internal control. The bar colors represent different time points of Cd stress. Data are presented as mean ± standard deviation (SD) from three biological replicates. Different lowercase letters above the bars indicate significant differences (*p* < 0.05) between time points for each gene. The figure was generated using GraphPad Prism 8. Significance analysis, denoted by letters, was conducted using one-way ANOVA followed by Tukey’s post hoc test with IBM SPSS Statistics 20.

**Figure 10:**
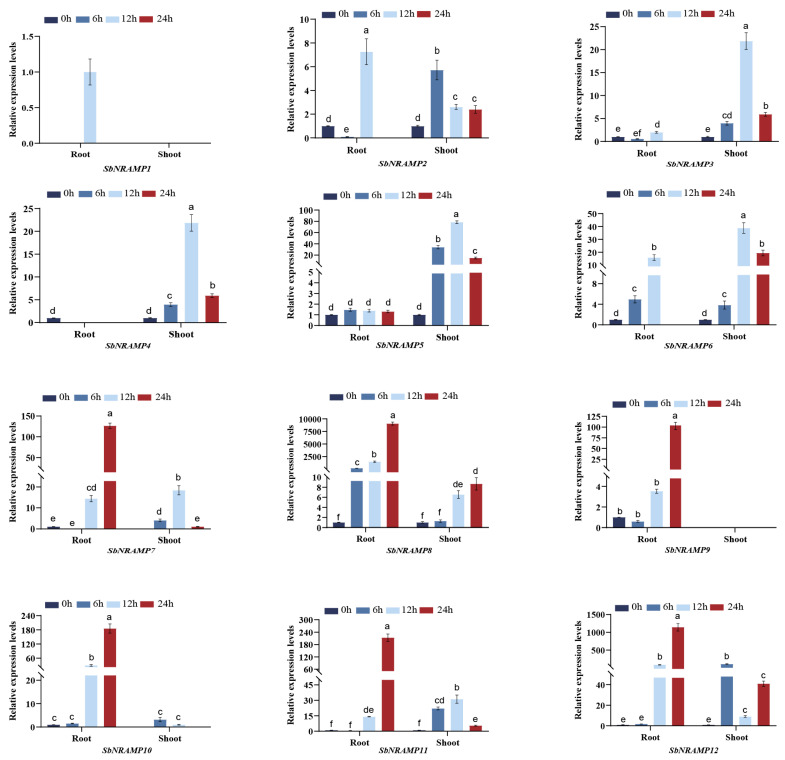
Expression patterns of *SbNRAMP* genes in sorghum seedling roots and shoots under manganese (Mn) stress. Transcript levels of *SbNRAMP* genes in two-leaf- and one-heart-stage sorghum seedlings after 6, 12, and 24 h of treatment with 100 μmol/L MnCl_2_·4H_2_O were analyzed. Gene expression levels were normalized using the sorghum *actin* gene (*SbACT*) as the internal control. The bar colors represent different time points of Mn stress. Data are presented as mean ± standard deviation (SD) from three biological replicates. Different lowercase letters above the bars indicate significant differences (*p* < 0.05) between time points for each gene. The figure was generated using GraphPad Prism 8. Significance analysis, denoted by letters, was conducted using one-way ANOVA followed by Tukey’s post hoc test with IBM SPSS Statistics 20.

**Figure 11:**
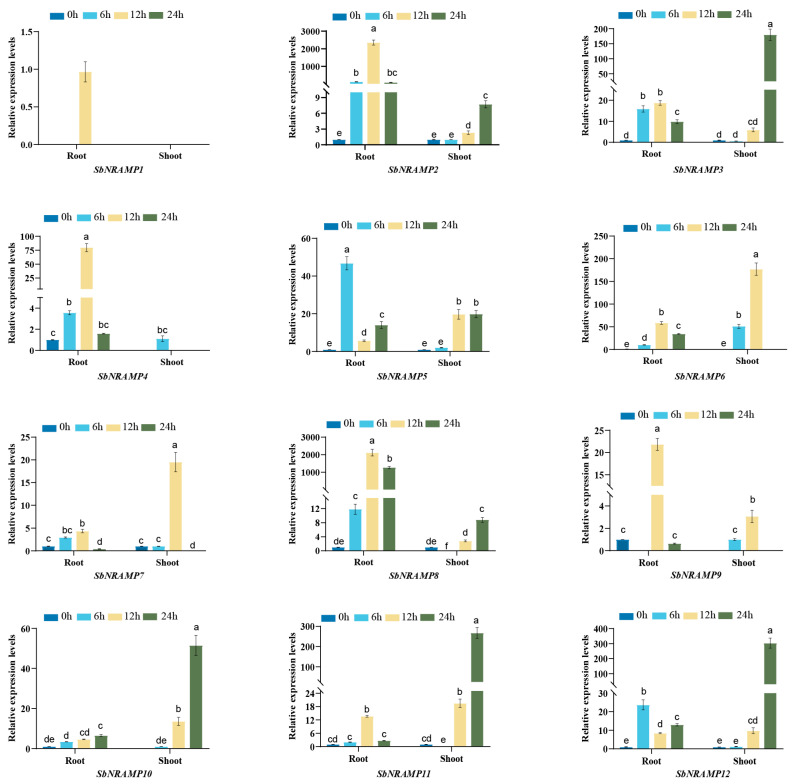
Expression patterns of *SbNRAMP* genes in sorghum seedling roots and shoots under zinc (Zn) stress. Transcript levels of *SbNRAMP* genes in two-leaf- and one-heart-stage sorghum seedlings after 6, 12, and 24 h of treatment with 100 μmol/L ZnCl_2_ were analyzed. Gene expression levels were normalized using the sorghum *actin* gene (*SbACT*) as the internal control. The bar colors represent different time points of Zn stress. Data are presented as mean ± standard deviation (SD) from three biological replicates. Different lowercase letters above the bars indicate significant differences (*p* < 0.05) between time points for each gene. The figure was generated using GraphPad Prism 8. Significance analysis, denoted by letters, was conducted using one-way ANOVA followed by Tukey’s post hoc test with IBM SPSS Statistics 20.

We analyzed the gene expression levels in the roots and shoots of sorghum seedlings under three different treatment conditions using quantitative reverse transcription polymerase chain reaction (qRT-PCR). The expression patterns of the 12 *SbNRAMP* genes in the roots and shoots exhibited significant differences (*p* < 0.05) when treated with Cd^2+^, Mn^2+^, or Zn^2+^ (Figure 9, Figure 10 and Figure 11). We normalized the gene expression values to the baseline at 0 h or, alternatively, based on the lowest expression observed at other time points when the genes were not expressed at 0 h. Utilizing the normalized data, we calculated the relative expression at subsequent time points. Notably, *SbNRAMP1* was detected at only a single time point in the roots under treatment with the three metals; specifically, it was observed in the roots treated with Cd for 24 h and in the roots treated with Mn and Zn for 12 h. The primary differences observed in the remaining genes are summarized as follows.

Under cadmium (Cd) stress (Figure 9), in the roots, Cd treatment alone markedly upregulated the expression of *SbNRAMP8*, *9*, and *10*. Additionally, *SbNRAMP2*, *5*, and *6* were significantly upregulated at both 12 and 24 h, while *SbNRAMP3* showed significant upregulation at 6 and 12 h. *SbNRAMP4* experienced significant upregulation at 12 h. Notably, *SbNRAMP7* was significantly upregulated at both 6 and 24 h but demonstrated a significant downregulation at 12 h. In contrast, *SbNRAMP11* expression did not exhibit significant changes, whereas *SbNRAMP12* was significantly upregulated at 6 and 24 h. In the shoots of sorghum seedlings, Cd treatment also led to a significant upregulation of *SbNRAMP2*, *5*, *6*, *11*, and *12* compared to the control group. *SbNRAMP3*, *4*, *8*, and *10* showed significant upregulation at 12 and 24 h. *SbNRAMP7* expression was not detected, and *SbNRAMP9* expression was only detected at 24 h.

Under manganese (Mn) stress (Figure 10), in the roots, Mn treatment alone resulted in a substantial upregulation of *SbNRAMP8*. The expression of *SbNRAMP2* showed a significant downregulation at 6 h, followed by a significant upregulation at 12 h. Additionally, *SbNRAMP3* was significantly upregulated at 12 h and *SbNRAMP9* was significantly upregulated at 24 h, while *SbNRAMP4* was not detected at any time points other than the control. *SbNRAMP5* exhibited no significant changes in expression. Conversely, *SbNRAMP6* was significantly upregulated at both 6 and 12 h. Furthermore, *SbNRAMP7*, *10*, *11*, and *12* were significantly upregulated at 12 and 24 h. In the shoots of sorghum seedlings, Mn treatment alone also led to a significant upregulation of *SbNRAMP2*, *3*, *4*, *5*, *6*, *10*, *11*, and *12*. Specifically, *SbNRAMP7* was significantly upregulated at both 6 and 12 h, while *SbNRAMP8* showed significant upregulation at 12 and 24 h. Notably, the expression of SbNRAMP9 was not detected.

Under zinc (Zn) stress (Figure 11), in the roots, Zn treatment alone led to a notable upregulation of *SbNRAMP2*, *3*, *4*, *5*, *6*, *8*, and *12*. Specifically, *SbNRAMP7* was significantly upregulated at 12 h but showed a significant downregulation at 24 h. Additionally, *SbNRAMP9* and *SbNRAMP11* were significantly upregulated at 12 h, while *SbNRAMP10* was significantly upregulated at 24 h. In the shoots of sorghum seedlings, Zn treatment alone resulted in significant upregulation of *SbNRAMP2*, *5*, *10*, *11*, and *12* at both 12 and 24 h compared to the control group. Furthermore, *SbNRAMP3* was significantly upregulated at 24 h, whereas *SbNRAMP4* expression was only detected at 6 h. *SbNRAMP6* and *SbNRAMP9* were significantly upregulated at both 6 and 12 h. Notably, *SbNRAMP7* was significantly upregulated at 12 h, with *SbNRAMP7* also showing significant downregulation at 24 h. *SbNRAMP8* demonstrated significant downregulation at 6 h, followed by significant upregulation at 24 h.

### 2.11. Effects of Metal Ions Stress on Physiological Indexes of Sorghum Seedlings

**Figure 12:**
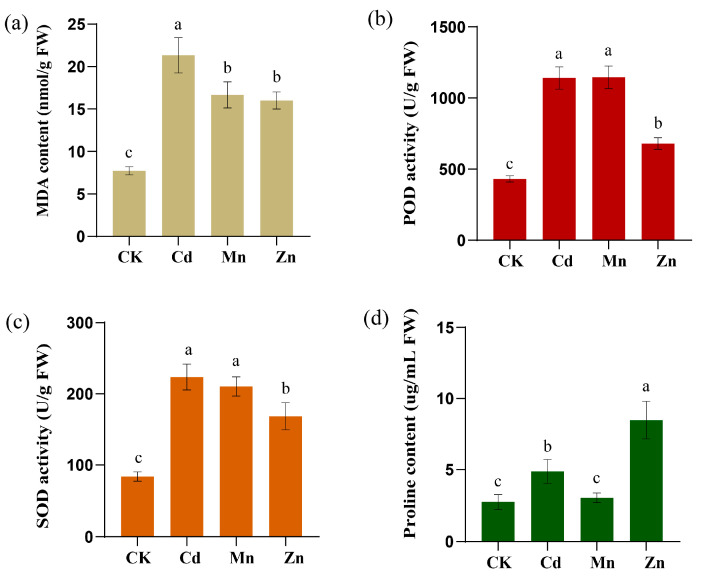
Determination of physiological indexes of sorghum seedlings under metal (Cd, Mn, or Zn) treatments. (**a**) represents malondialdehyde (MDA) content. (**b**) represents peroxidase (POD) activity. (**c**) represents superoxide dismutase (SOD) activity. (**d**) represents proline (Pro) content. Data are presented as mean ± standard deviation (SD) from three biological replicates. Different lowercase letters (a–c) above the bars within each panel indicate significant differences (*p* < 0.05) among the different metal treatments for that specific physiological index. Statistical significance was determined using one-way ANOVA followed by Tukey’s post hoc test (IBM SPSS Statistics 20). The figure was generated using GraphPad Prism 8.

The concentrations of stress-related metabolites and changes in the activities of antioxidant enzymes reflect the stress conditions experienced by plants. This study investigates the effects of exposure to three heavy metals (100 μmol/L MnCl_2_·4H_2_O, 100 μmol/L CdCl_2_, or 100 μmol/L ZnCl_2_) on sorghum seedlings at the two-leaf and one-heart stage over a period of two days (Figure 12).

The measurement of malondialdehyde (MDA) content in sorghum seedlings indicated that MDA levels significantly increased under all three metal treatments (Figure 12a), with the highest levels observed under Cd^2+^ treatment.

We observed that the activities of POD (Figure 12b) and SOD (Figure 12c) in the Cd^2+^, Mn^2+^, and Zn^2+^ treatment groups were significantly higher than those in the control group. The activities of POD and SOD were higher following treatment with Cd^2+^ and Mn^2+^ than those for Zn^2+^.

Furthermore, the determination of proline (Pro) content revealed that Pro levels for the Cd^2+^ and Zn^2+^ treatments increased (Figure 12d), with the most significant increase noted in the Zn^2+^ treatment group, while the Pro content in the Mn^2+^ treatment group did not show a significant increase. This indicates that the three different metal stresses exert varying effects on the growth and development of sorghum seedlings.

### 2.12. SbNRAMP6 Was Localized in the Cell Membrane

**Figure 13:**
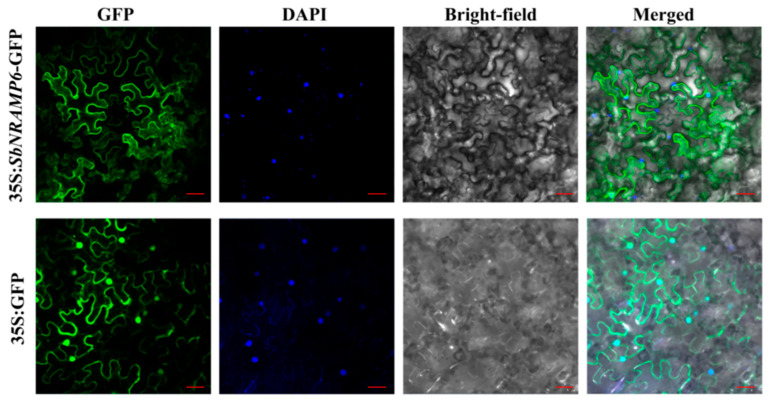
SbNRAMP6 is localized to the membrane. The 35S:*SbNRAMP6*–GFP fusion protein was transiently expressed in tobacco. Nuclei are counterstained with DAPI (blue). Bright-field images show cell morphology, and merged images combine GFP, DAPI, and bright-field channels. Scale bar = 20 µm.

Subcellular localization predictions using Plant-mPLoc indicated that all *SbNRAMP* genes are localized to the plasma membrane and two of them are also located in other compartments (the chloroplast and nucleus) (Table 2). To experimentally validate these predictions, a subcellular localization analysis was conducted on *SbNRAMP6* in *Nicotiana benthamiana*. The PBI121–*SbNRAMP6*–GFP expression vector was successfully constructed, and fluorescence microscopy revealed distinct green fluorescence, confirming effective expression in tobacco (Figure 13). Notably, GFP signals were enriched in the plasma membrane, exhibiting only partial overlap with DAPI staining, a nuclear marker. These findings align with bioinformatic predictions, further supporting the localization of *SbNRAMP6* to the plasma membrane.

## 3. Discussion

The Natural Resistance-Associated Macrophage Protein (NRAMP) family is a highly conserved family of metal ion transporters that are widely found in various organisms [30,31]. The *NRAMP* gene family has been reported in many plant species, primarily responsible for the absorption, transport, and maintenance of intracellular ion balance, such as Fe, Cd, Mn, and Zn [32]. Previous studies have identified members of the *NRAMP* gene family in the genomes of several species, including *Oryza sativa* [33], *Zea mays* [18], *Arabidopsis thaliana* [21], *Areca catechu* [34], *Camellia sinensis* [35], and *Hibiscus cannabinus* [36]. In this study, we identified 12 *NRAMP* genes from the sorghum genome (Figure 1). Based on their chromosomal locations, we designated each gene individually. Notably, previous studies classified the seven *NRAMP* genes in rice into two subfamilies [33]; here, we categorized the twelve members identified into three subfamilies (Figure 3), based on their genetic relationships and structural characteristics within the *SbNRAMP* gene family. The unique structures of *SbNRAMP3* and *SbNRAMP8* in the third subfamily suggest that *NRAMP* genes in sorghum may have evolved novel functions, including specialized metal transport and stress-responsive regulation.

The analysis of physicochemical properties is essential for understanding the potential functional characteristics of proteins [37]. Similarly to other members of the *NRAMP* gene family in various plant species, the 12 SbNRAMPs comprise both acidic (pI ranging from 4.92 to 5.42) and alkaline (pI ranging from 8.62 to 8.93) proteins [38]. Most SbNRAMPs (9 out of 12) exhibit an instability coefficient of less than 40, indicating that SbNRAMP is considered a stable protein [16]. The amino acid lengths and molecular weights of the majority of SbNRAMPs (10 out of 12) are relatively consistent, ranging from 466 to 559 amino acids with a molecular weight of 50.75 to 60.15 kDa. Notably, the amino acid lengths and molecular weights of SbNRAMP3 and SbNRAMP8 exceed those of other members by more than double (Table 1). This suggests that *SbNRAMP3* and *SbNRAMP8* may perform unique functions due to their distinct physicochemical properties. Interestingly, the expression levels of *SbNRAMP3* and *SbNRAMP8* varied significantly under three different metal stresses. *SbNRAMP8* exhibited the highest expression in the roots of sorghum seedlings, whereas *SbNRAMP3* showed the highest expression in the shoots of these seedlings (Figure 9, Figure 10 and Figure 11). Numerous previous studies have demonstrated that *NRAMP* gene family members can perform distinct functions across various organelles. For instance, in *A. thaliana*, AtNRAMP6 is localized to the Golgi/trans-Golgi network and endosomal compartments, where it plays a crucial role in maintaining intracellular iron homeostasis and influences the growth of lateral roots under conditions of iron deficiency [39,40]; AtNRAMP3 and AtNRAMP4 are both located in the vacuolar membrane [41]. Under conditions of iron deficiency, these transporters are transcriptionally upregulated to facilitate the remobilization of vacuolar Mn^2+^ reserves into the cytosol. This mechanism is essential for maintaining Mn^2+^ homeostasis, which is critical for the function of photosystem II during seed germination and manganese deficiency stress. Additionally, these transporters regulate the vacuolar efflux of divalent metal ions, including Mn^2+^ and Fe^2+^, thereby modulating the availability of cytoplasmic metals, which ultimately influences ion distribution in both leaves and roots [5,42]. For example, AtNRAMP1 is localized to the plasma membrane [43]. The expression of *AtNRAMP1* is upregulated in response to iron deficiency in roots. In rice, most OsNRAMPs are also plasma membrane-localized and involved in ion balance [44,45]. OsNRAMP1 is localized to the plasma membrane and facilitates the uptake of cadmium (Cd). Its primary physiological role is to maintain the homeostasis of manganese (Mn^2+^) and iron (Fe^2+^) [46]. Similarly localized to the plasma membrane, OsNRAMP5 serves as a primary transporter for manganese (Mn^2+^) and iron (Fe^2+^), while also facilitating the uptake of cadmium (Cd) [47,48], facilitating the movement of these ions from the roots to the shoots [49,50]. It is predicted that SbNRAMP3 is localized in the plasma membrane and nucleus, whereas SbNRAMP8 is anticipated to be present in both the plasma membrane and chloroplasts (Table 2). Meanwhile, the tissue expression of *SbNRAMP3* and *SbNRAMP8* exhibited distinct specificity. *SbNRAMP8* was highly expressed across 11 tissues, with the highest levels observed in the floral meristem and vegetative meristem. In contrast, *SbNRAMP3* demonstrated high expression in six tissues, predominantly in anther and plant embryos (Figure 6). Consequently, we hypothesize that the observed differences in tissue expression may arise from distinct predictions of subcellular localization. However, the specific roles of *SbNRAMP3* and *SbNRAMP8* in regulating metal ion homeostasis require further investigation.

Dynamic variations in the lengths of the 5′ untranslated region (UTR) and 3′ UTR act as *cis*-regulatory mechanisms that finely regulate stress-responsive genes’ expression in plants by modulating transcript stability and translational efficiency [51,52]. Structural analysis revealed that SbNRAMP4 and SbNRAMP2 proteins lack both 5′ and 3′UTRs, while SbNRAMP11 lacks the 5′UTR (Figure 3b). The presence of a 5′UTR is associated with increased RNA and protein accumulation [53]. SbNRAMP8 and SbNRAMP12 exhibit long 5′UTR, SbNRAMP1 has an extended 3′UTR, and SbNRAMP10 is distinguished by a high intron number. The gene structure characteristics of these pre-mRNA structures are worthy of further study and discussion. Given that intron length is inversely correlated with gene expression [54,55], the large intron count in SbNRAMP10 likely results in low expression efficiency without contributing to an increase in protein length.

Subcellular localization analysis predicted that most of the SbNRAMP genes are situated in the plasma membrane (Table 2). Subsequently, based on the integrity of the protein structure, specifically the complete intron–exon arrangement, we categorized tissue expression into high expression levels, defined as expression in at least two tissues. Under the stress of three metals (Cd, Mn, or Zn), significant expression (*p* < 0.05) was observed in both the roots and shoots. The gene of interest, *SbNRAMP6*, was successfully cloned, and subcellular localization analysis confirmed that its targeting pattern aligns with bioinformatic predictions; SbNRAMP6 localizes to the plasma membrane (Figure 13). Current studies indicate that most NRAMPs are located on the plasma membrane [18,19]. Given the membrane localization and the conservation of the protein sequence of SbNRAMP6 (Figure A2), we propose that this protein facilitates the transmembrane transport of metal ions, such as Cd, Mn, and Zn, similarly to other members of the *NRAMP* family, thereby regulating ion homeostasis and stress responses in plants [56,57].

All SbNRAMPs share motifs 3, 5, 9, and 10, highlighting strong conservation and suggesting functional similarity (Figure 3c). Promoter analysis results showed that *SbNRAMP5* and *SbNRAMP10* contain ABREs, indicating abscisic acid regulation, while *SbNRAMP1* is enriched with CGTCA motifs, suggesting responsiveness to methyl jasmonate (Figure 4). The promoters of many *SbNRAMP* genes possess defense and stress response *cis*-acting elements, highlighting their roles in plant defense and adaptation. Through WGCNA of transcriptome data, we identified *SbNRAMP5* as a key gene associated with saline–alkali stress in the roots of sorghum seedlings. Additionally, *SbNRAMP5* was found to be highly expressed in a tissue-specific manner. In the roots, flowers, and shoots of plants, the expression is most significant (Figure 6), exhibiting significant expression across multiple tissues. We hypothesize that *SbNRAMP5* may play a crucial role in abiotic stress response; however, the specific functions of this gene require further investigation.

Previous studies have demonstrated that *NRAMPs* are associated with the uptake and transport of various metal ions in plants [19]. In *A. thaliana*, the gene *AtNRAMP2* exhibits manganese transport activity, facilitating the entry of manganese into the cytoplasm of yeast and effectively rescuing the manganese deficiency phenotype [58]. Additionally, *AtNRAMP1* serves a dual role in the absorption and transport of both manganese and iron, playing a crucial role in regulating iron uptake in roots. It is also identified as a key transporter for manganese absorption, particularly under conditions of low manganese availability [43]. In rice, there are seven *NRAMP* genes. *OsNRAMP3* serves as a regulator of manganese distribution in plant tissues [45]. *OsNRAMP5* serves as the primary transporter for manganese (Mn^2+^) uptake from soil by plant roots, while also facilitating the secondary accumulation of cadmium (Cd) [49,59]. *OsNRAMP4* is the first transporter recognized as a trivalent aluminum ion transporter within this family; however, its protein structure exhibits less similarity with other family members, indicating that *OsNRAMP4* is not involved in the transport of metals such as zinc, manganese, and iron [60]. In this study, we analyzed the expression profiles of the *SbNRAMP* genes in the aboveground parts and roots of sorghum seedlings subjected to three metal treatments. Our findings indicate that, under the stress of these three metals, *SbNRAMP1* exhibits a transient induction that occurs exclusively at specific time points under defined metal stresses (Cd stress at 24 h, Mn and Zn stress at 12 h), while other family members show sustained differential expression across multiple time points. (Figure 9, Figure 10 and Figure 11). This suggests that the *SbNRAMP* gene family may play a crucial role in the transport of metals in both the shoots and roots of sorghum seedlings, with marked differences in the expression of various genes.

Through the assessment of physiological indices under stress, we observed that, compared to the control group, the activity of key antioxidant enzymes in the treatment group (Figure 12)—including superoxide dismutase (SOD) and peroxidase (POD)—increased significantly. This finding suggests that sorghum mitigated metal ion stress by modulating its antioxidant enzyme system [61]. Malondialdehyde (MDA) is a significant end product of lipid peroxidation in plant membranes, particularly under heavy metal stress [62,63]. The accumulation of MDA in plants can lead to severe damage to cell membranes [64]. Our results indicate that the content of MDA, a biomarker for oxidative damage, increased significantly, suggesting that the membrane system of sorghum seedlings was compromised under the stress of three different metals. These physiological changes indicate that sorghum seedlings can mitigate the damage caused by reactive oxygen species by regulating their own antioxidant enzyme systems when exposed to metal stress.

## 4. Materials and Methods

### 4.1. Material Cultivation and Treatment

*Sorghum bicolor* seeds were surface-sterilized with 0.1% H_2_O_2_ for 10 min and subsequently rinsed four times with distilled water until the wash was clear. The seeds were then placed on filter paper in Petri dishes and incubated at 23 °C under a 16-h light/8-h dark cycle in a greenhouse to promote germination. Once the seedlings reached approximately 25 mm in height, they were transferred to hydroponic boxes containing half-strength Hoagland solution. Upon reaching the two-leaf and one-heart stage, uniformly growing seedlings were selected for metal stress experiments. The seedlings were divided into control and treatment groups, with the control group maintained in half-strength Hoagland medium, while the treatment group received half-strength Hoagland medium supplemented with 100 μmol/L MnCl_2_·4H_2_O, 100 μmol/L CdCl_2_, or 100 μmol/L ZnCl_2_ [65]. Samples from aboveground tissues and roots were collected at 0 h for the control group and at 6, 12, and 24 h for the treatment group, with three replicates per condition. Each replicate included five seedlings, and 0.1 g of each tissue type was harvested per replicate. Collected shoots and roots were immediately frozen in liquid nitrogen and stored at −80 °C for subsequent RNA extraction.

### 4.2. Total RNA Extraction, cDNA Synthesis, and NRAMP Gene Expression Analyses

At each time point, 0.1 g of root and shoot tissues were collected from sorghum seedlings. Total RNA was isolated from the shoots and roots using the TRIzol reagent kit (Shanghai Huiying Biotechnology Co., Ltd., Shanghai, China), and first-strand cDNA was synthesized with the Thermo Scientific (Waltham, MA, USA) RNA reverse transcription kit. The resulting cDNA served as the template for quantitative real-time PCR (qRT-PCR). Twelve primer pairs were designed for qRT-PCR in accordance with standard primer design criteria. Actin (Gene ID: LOC110436378, NCBI) was utilized as the internal reference gene. Each 20 μL qRT-PCR reaction comprised 10 μL of 2× SYBR Green Master Mix (Tiangen, Beijing, China), 1 μL each of forward and reverse primers, 1 μL of cDNA, and 7 μL of deionized water. The amplification protocol included initial denaturation at 94 °C for 30 s, followed by 40 cycles of denaturation at 94 °C for 30 s and annealing/extension at 60 °C for 30 s. The expression of 12 *SbNRAMP* genes was quantified using gene-specific primers, and relative expression levels were calculated via the 2^−ΔΔCt^ method [66]. All experiments were conducted in triplicate. All primers used in this study are listed in Table A1.

### 4.3. Determination of Physiological Indexes

Sorghum seedlings were treated with half-strength Hoagland medium supplemented with 100 μmol/L MnCl_2_·4H_2_O, 100 μmol/L CdCl_2_, or 100 μmol/L ZnCl_2_ for 2 days. The control group was cultured in half-strength Hoagland medium for the same period. The aboveground portions (sheet) of the sorghum seedlings were harvested and rapidly frozen in liquid nitrogen. Each treatment group consisted of three replicates, each containing four sorghum seedlings. Subsequently, the activities of antioxidant enzymes were measured, with a focus on superoxide dismutase (SOD), peroxidase (POD), malondialdehyde (MDA), and proline (Pro). All substances were quantified using a test kit from Beijing Solebold (Beijing, China), which provides detailed information regarding the experimental procedures.

### 4.4. Subcellular Localization Analysis

The construction of the pB121–*SbNRAMP6* vector was based on the published method [67]. *Agrobacterium tumefaciens* (GV3101) was used for the transient transformation of the 35S:*SbNRAMP6*–green fluorescent protein (*SbNRAMP6*-GFP) vector and the 35S:GFP empty vector, which were injected into the epidermal cells of 4-week-old tobacco plants [68]. Transgenic tobacco was initially cultured in the dark for one day, followed by normal cultivation for two additional days. Subsequently, a 1 μg/mL DAPI solution was injected into the leaves and incubated in the dark for 20 min. The blade was cut into 1 cm^2^ pieces to make temporary slides. The fluorescence signal was observed using a laser scanning confocal microscope (Leica; Wetzlar, Hessen, Germany).

### 4.5. Identification and Physicochemical Properties Analysis of SbNRAMP

The sorghum genome, protein, GFF3 annotation, and coding sequence (CDS) data were downloaded from the NCBI database (https://www.ncbi.nlm.nih.gov/). Six *NRAMP* gene sequences from *A. thaliana* were obtained from the TAIR database (https://www.arabidopsis.org/). The NRAMP sequences of *A. thaliana* and sorghum were aligned using Tbtools to preliminarily identify NRAMP candidates based on sequence similarity. The hidden Markov model (HMM) file for NRAMP (PF01566) was retrieved from the Pfam database (http://pfam-legacy.xfam.org/), and HMMER v3.0 was employed for sequence retrieval using the hmmsearch program. Sorghum protein sequences with E-values below 0.05 were retained as additional candidates. Overlapping results from the two identification methods were compared to eliminate duplicates. Candidate sequences were further validated using the Ensembl Plants database (https://plants.ensembl.org/index.html, accessed on 11 October 2024), and the final NRAMP family members were confirmed based on the presence of two conserved motifs. The characteristics of the 12 identified SbNRAMPs, including amino acid number, molecular weight, isoelectric point, atom count, instability index, aliphatic index, and hydrophilicity, were predicted using the ProtParam tool on the ExPASy platform (https://www.expasy.org/).

### 4.6. Chromosomal Localization, Protein Structure, and Subcellular Localization Prediction

Chromosome density files were generated from the whole-genome annotation of sorghum using TBtools. Target genes were identified and visually pre-mapped within TBtools by integrating the protein sequence file, genome annotation, and chromosome density data. Gene annotation information for the 12 *SbNRAMP* genes was extracted from the genome annotation, and the visualization function of TBtools was employed to display their chromosomal locations.

The secondary structures of the 12 NRAMPs were predicted using SOPMA, while their tertiary structures were modeled through comparative modeling with SWISS-MODEL (https://swissmodel.expasy.org/). Subcellular localization for all 12 NRAMPs was determined using the Plant-mPLoc (http://www.csbio.sjtu.edu.cn/bioinf/plant-multi/, accessed on 15 April 2025) server.

### 4.7. Multiple Sequence Alignment and Phylogenetic Analysis

The protein sequence alignment of SbNRAMP was conducted using MEGA 11.0, and the resulting FASTA file was subsequently visualized using GeneDoc 2.7 software. The frequency of conserved protein sequences was plotted using the WebLogo 3 online tool (https://weblogo.berkeley.edu/logo.cgi, accessed on 11 March 2025).

A phylogenetic tree representing the NRAMP gene family across multiple species was constructed. Species data were sourced from NCBI, and the corresponding protein sequences were retrieved using TBtools v2.085 software. Phylogenetic trees for the NRAMP gene family members across sorghum, rice, *A. thaliana*, foxtail millet, and maize were generated using MEGA 11.0 software. The bootstrap value was set to 1000 repetitions, employing the neighbor-joining (NJ) method, and the online platform ITOL (https://itol.embl.de/, accessed on 14 April 2025) was utilized to enhance the visual presentation of the resulting phylogenetic tree.

### 4.8. Conserved Domain Analysis of SbNRAMPs

The protein sequences of the SbNRAMP family were submitted to TBtools software for analysis. The number of motifs was set to 10, while the remaining parameters remained unchanged. The results were visualized using TBtools’ built-in software. Each motif sequence was identified and saved using the MEME v5.5.8 online software (https://meme-suite.org/meme/, accessed on 15 April 2025).

### 4.9. Analysis of Cis-Acting Elements of SbNRAMP Promoter

The promoter sequences of the SbNRAMPs were extracted using TBtools v2.085 software and subsequently submitted to the online tool Plant CARE v1.0 (https://bioinformatics.psb.ugent.be/webtools/plantcare/html/, accessed on 3 May 2025) for analysis. The obtained data were screened and analyzed, and the final results were visualized using TBtools software to create a *cis*-acting element map.

### 4.10. Replication Events Between SbNRAMPs

The chromosomal location of the *SbNRAMP* genes can be determined using the sorghum genome annotation file. Additionally, the collinearity information for the *SbNRAMP* genes can be extracted using TBtools software, while the collinearity relationships of the *SbNRAMP* genes were visualized with the Circos program within TBtools.

### 4.11. SbNRAMP Expression Profile Mapping

The FPKM expression values of the *SbNRAMP* genes across various tissues were obtained utilizing the Sorghum Functional Genomics Database (http://structuralbiology.cau.edu.cn/sorghum/index.html, accessed on 22 October 2024) for online analysis. This dataset includes expression information for various tissues, such as floral meristem, flower, root, shoot, vegetative meristem, leaf, early inflorescence, inflorescence, 5-day and 10-day post-pollination seed, anther, pistil, plant embryo, and endosperm tissue. Subsequently, a heatmap of the expression data was generated using TBtools software. The expression levels were calculated using log_2_ ^FPKM^ [69].

### 4.12. Weighted Gene Co-Expression Network Analysis (WGCNA)

The transcriptome data utilized in this study were derived from previously published experimental studies. Sorghum seedlings, characterized by the presence of the third true leaf, were treated with a modified Hoagland solution containing 150 mM NaHCO_3_. The aboveground parts and roots of both the control group (0 h) and treatment groups (NaHCO_3_ treatment at 6, 12, and 24 h) were randomly collected for RNA extraction [70]. The phrase ‘randomly collected’ signifies that samples were taken from multiple seedlings in each replicate, without bias towards specific parts (beyond being aboveground). This approach ensures that the samples are pooled to accurately represent the average shoot response. The gene expression matrix was generated based on the expression levels of genes under saline–alkali stress at different time points (0 h, 6 h, 12 h, and 24 h). A Weighted Gene Co-expression Network Analysis (WGCNA) network was constructed using a threshold of 0.5-fold and a minimum of 30 gene counts to integrate closely related genes into distinct modules. Data analysis was performed using the WGCNA-shiny plugin in TBtools (https://github.com/ShawnWx2019/WGCNA-shinyApp, accessed on 12 November 2024). Modules that displayed a correlation coefficient of ≥0.8 and a *p*-value of ≤0.05 were designated as stress-related modules.

### 4.13. Statistical Analysis

Experimental data were processed and analyzed using Microsoft Excel 2019 for data organization, IBM SPSS Statistics 20 for statistical analysis, and GraphPad Prism 8 for visualization and graphing. Tukey’s post hoc test was applied for multiple comparisons in ANOVA, with a significance level set at *p* < 0.05.

## 5. Conclusions

In this study, we systematically identified 12 *NRAMP* genes from the whole genome of two-color sorghum. These *SbNRAMP* genes are randomly distributed across seven chromosomes of sorghum, containing two pairs of fragment repeats, and are further classified into three families (I-III). The three subfamilies exhibit certain differences in conserved motifs and protein structures, with members within a single subfamily showing high similarity. These SbNRAMPs exhibit tissue specificity and functional diversity throughout growth and development. They play a crucial role in regulating plant responses to metal ion stress, particularly in response to cadmium (Cd), manganese (Mn), and zinc (Zn). This study provides a foundational and theoretical basis for investigating the functions and mechanisms of the sorghum gene family in plant growth and development.

## Figures and Tables

**Table 1:** Physicochemical properties of sorghum *NRAMP* family proteins. ^a^ amino acid number; ^b^ molecular weight; ^c^ grand average of hydropathicity; ^d^ isoelectric points; ^atoms^ total number of atoms. The protein sequences of the twelve SbNRAMPs were obtained either from the sorghum genome or retrieved from UniProt using their respective protein IDs.

Gene Name	Protein ID	Aa ^a^	MW ^b^ kDa	pI ^d^	N_atoms_	Instability	Aliphatic Index	GRAVY ^c^	Exons	Introns
*SbNRAMP1*	A0A1B6QIP3	466	50.75	7.16	7281	26.38	127.34	0.83	6	5
*SbNRAMP2*	A0A1Z5S5F1	486	53.06	5.99	7533	33.4	116.98	0.622	5	4
*SbNRAMP3*	A0A1B6QIN8	1236	134.71	6.02	18,946	41.62	94.73	0.048	7	6
*SbNRAMP4*	A0A1B6QJE9	546	59.95	5.57	8509	38.01	107.91	0.441	4	3
*SbNRAMP5*	C5WTQ4	516	56.35	6.17	8048	34.24	114.38	0.546	4	3
*SbNRAMP6*	A0A1W0W334	535	58.38	7.07	8383	38.33	121.4	0.569	12	11
*SbNRAMP7*	C5 × 2P7	525	56.37	6.74	8097	38.77	127.41	0.767	13	12
*SbNRAMP8*	C5XKD6	1157	125.88	6.07	17,699	47.16	91.21	−0.036	5	5
*SbNRAMP9*	A0A194YMG4	547	59.39	6.03	8478	32.7	114.28	0.576	13	12
*SbNRAMP10*	A0A1Z5RJE5	559	60.15	8.52	8614	43.34	118.28	0.68	13	13
*SbNRAMP11*	C5YQG7	544	58.89	4.89	8376	32.8	111.23	0.469	4	3
*SbNRAMP12*	C5Z7T5	550	59.49	8.45	8521	32.69	118.95	0.578	13	12

**Table 2:** SbNRAMP transmembrane domain number, subcellular localization prediction, and secondary structure prediction information, including alpha helix, extended strand, beta turn, and random coil. Protein secondary structures (SOPMA) and subcellular localization (Plant-mPLoc) were computationally predicted.

Protein Name	TM Domains	Subcellular Localization	Alpha Helix (%)	Extended Strand (%)	Beta Turn (%)	Random Coil (%)
SbNRAMP1	11	Plasma membrane	53.86	16.95	2.58	26.61
SbNRAMP2	10	Plasma membrane	55.97	12.76	2.88	28.40
SbNRAMP3	10	Plasma membrane. nucleus	38.35	13.35	3.16	45.15
SbNRAMP4	10	Plasma membrane	56.23	11.72	2.93	29.12
SbNRAMP5	9	Plasma membrane	59.11	10.47	3.49	26.94
SbNRAMP6	11	Plasma membrane	52.90	12.71	2.99	31.40
SbNRAMP7	11	Plasma membrane	54.29	13.52	3.24	28.95
SbNRAMP8	9	Plasma membrane. Chloroplast	35.52	11.67	2.59	50.22
SbNRAMP9	10	Plasma membrane	57.04	12.98	3.29	26.69
SbNRAMP10	10	Plasma membrane	58.50	11.63	3.58	26.30
SbNRAMP11	10	Plasma membrane	58.27	12.68	2.94	26.10
SbNRAMP12	12	Plasma membrane	53.09%	13.64%	2.91	30.36

## Data Availability

The data presented in this study are available in the European Molecular Biology Laboratory’s (EMBL’s) European Bioinformatics Institute database at [https://www.embl.org/], reference numbers E-MTAB-5956, E-MTAB-4273, E-GEOD-98817, and E-CURD-25. (SUB13694414).

## Appendix A

**Table A1:** Primers for qRT-PCR analysis in this study.

Gene Name	Primer Name	Primer Sequence (5′–3′)	Tm (°C)	Length (bp)
*SbNRAMP1*	F	AGCACTCGTGGTCTCAATGG	60	193
	R	ACTCCTGGTGGCAAATCTCG		
*SbNRAMP2*	F	ACCAGCAAGATAACCGAGGC	60	184
	R	TCTTTCAGGAACAGCAGCGT		
*SbNRAMP3*	F	GGCTGTTTCGTCAGAATGGC	59	157
	R	CAGTGAAGCCTGCCAAACAC		
*SbNRAMP4*	F	TCCACCCACCTATGAAGCCGA	62	181
	R	CTGAGGCATAGAAGGCACCCT		
*SbNRAMP5*	F	GCGCTCGATTGCTTCATCTT	59	175
	R	TGATGCAGCCCACAATTCCA		
*SbNRAMP6*	F	TCGCAGAGGTGGCTGTAATC	60	119
	R	CTTCCGCACCCCGTATCTTT		
*SbNRAMP7*	F	GGTGTAGCAATGTTCGTGGC	60	198
	R	TTGCCCAGCGTAAGTACCAG		
*SbNRAMP8*	F	ATATCTTCTCGGCGCAGTCG	60	208
	R	TTGAACAGCCACCCTGATCC		
*SbNRAMP9*	F	AATGCAGACAACCTCTCCCC	59	140
	R	ATGATGACCTGCCCAGCAAA		
*SbNRAMP10*	F	TGTGGAGCAAGCACTTTCCT	60	148
	R	TGGCATAAACCCCTCGCAAT		
*SbNRAMP11*	F	GCCCTAACACCCAAGCTGTA	59	175
	R	CTGCTGTGGACTGCTTTGAC		
*SbNRAMP12*	F	TGTCCACCGTCTCCCAAGTA	60	166
	R	ATGTCCTCTCGGGGCAAATG		
